# Supplementary material for: Hetero-site nucleation for growing twisted bilayer graphene with a wide range of twist angles
Source: Nat Commun. 2021 Apr 22;12:2391. doi: 10.1038/s41467-021-22533-1 (PMC8062483; doi:10.1038/s41467-021-22533-1)
Supplement: Supplementary file 1 — Supplementary Information [file 41467_2021_22533_MOESM1_ESM.pdf]

Supplementary information for:

Hetero-site nucleation for growing twisted bilayer graphene with a wide range of twist angles

Luzhao Sun[1,2,3,†], Zihao Wang[4,†], Yuechen Wang[1,2,†], Liang Zhao[5], Yanglizhi Li[1,2,3], Buhang Chen[3], Shenghong Huang[6]*, Shishu Zhang[1], Wendong Wang[4], Ding Pei[7], Hongwei Fang[8], Shan Zhong[1], Haiyang Liu[1], Jincan Zhang[1,3], Lianming Tong[1], Yulin Chen[7,8], Zhenyu Li[9], Mark H. Rümmeli[5], Kostya S. Novoselov[4], Hailin Peng[1,3]*, Li Lin[4]* & Zhongfan Liu[1,3]*.

[1] Center for Nanochemistry, Beijing Science and Engineering Center for Nanocarbons, Beijing National Laboratory for Molecular Sciences, College of Chemistry and Molecular Engineering, Peking University, Beijing 100871, P. R. China.

[2] Academy for Advanced Interdisciplinary Studies, Peking University, Beijing 100871, P. R. China.

[3] Beijing Graphene Institute, Beijing 100095, P. R. China.

[4] School of Physics and Astronomy, University of Manchester, Manchester M13 9PL, UK.

[5] Soochow Institute for Energy and Materials Innovation, Soochow University, Suzhou 215006, P. R. China.

[6] Department of Modern Mechanics, Unifersity of Science and Technology of China, Hefei, 230026, P. R. China.

[7] Clarendon Laboratory, Department of Physics, University of Oxford, Parks Road, Oxford OX1 3PU, UK.

[8] School of Physical Science and Technology, ShanghaiTech University, Shanghai 201210, P. R. China

[9] Hefei National Laboratory for Physical Sciences at the Microscale, University of Science and Technology of China, Hefei, 230026, P. R. China.

[†] *These authors contributed equally to this work*

*Correspondence should be addressed to Z.F. Liu (zfliu@pku.edu.cn), L. Lin (linli-cnc@pku.edu.cn), H.L. Peng (hlpeng@pku.edu.cn), and S.H. Huang (hshnpu@ustc.edu.cn).

This supplementary information includes:

Supplementary notes 1-8

Supplementary Tables 1-3

Supplementary Figs. 1-27

Supplementary References (1-24)

Supplementary Note 1: Growth modes of bilayer graphene and the factors that influence stacking order

The growth of bilayer graphene or few-layer graphene (FLG) follow one of two modes: 1) **Mode-I**, the secondary-layer islands grow on the top layer of the first-layer graphene; 2) **Mode-II**, the secondary-layer islands grow beneath the first layer, in which the growth behavior is strongly influenced by the Cu substrate. Generally, if the carbon-source supply is extremely high, the growth of secondary-layer graphene would follow the first model, with irregular morphologies. If the carbon source supply is low or partial pressure of H_2 is relatively high, the graphene edge is usually terminated by hydrogen, and the decomposed carbon atoms can diffuse underneath the first layer graphene. In this case, considering the etching effect of hydrogen, the as-received graphene domains usually exhibit regular hexagonal shape.

The orientation of graphene is determined at the nucleation stage, which is influenced by the chemical environment surrounding to the nuclei[1,2]. According to the growth mode-II, the second layer graphene nucleates and grows near the substrate, indicating that the substrate plays crucial roles in determining the orientation of the second layer graphene. If the two graphene layers are formed simultaneously, they will prefer to choose the same orientation, as AB stacking without any interlayer rotation provides the most stable configuration (Supplementary Fig. 1a). If the second layer is formed after the nucleation of first layer, the orientation of the second layer is mainly influenced by the underlying Cu substrate. Especially, the interactions between the graphene edge and Cu atoms are more important than those between graphene layers[3]. Therefore, the orientations of the second layers are determined mainly by the microscopic environment of Cu substrate near their nucleation sites in our hetero-site nucleation strategy, which are clearly different from those of the first layer (Supplementary Fig. 1b).

Supplementary Note 2: Raman spectra of tBLGs

Raman spectra were acquired in tBLGs samples with the twist angles in the full range from 0° to 30°, confirming interlayer rotation in as-received tBLGs. An excitation wavelength of 532 nm (corresponding to photon energy of 2.33 eV) was used, which can produce the rotation-related R' peak for BLGs with small twist angles (<10°) due to intravalley double-resonance. R' peak is

nondispersive, whose maximum intensity occurs at the critical angle where the excited phonons match the laser energy[4,5]. Supplementary Fig. 2a shows the R' peaks of tBLG with twist angle of 3° (3°-tBLG), 6° (6°-tBLG), and 9° (9°-tBLG). The intensity of R' peak of 6°-tBLG is higher than those of 3°-tBLG and 9°-tBLG, which agrees well with the reported literature[4-6]. Fig. 1c and Supplementary Fig. 2a also show typical spectra of 12°-tBLG, in which the G band is strongly enhanced and a new twist-related R peak is clearly observed. The enhancement of G band is ascribed to the energy matching between the van Hove singularities (vHSs) and exciting laser (2.33 eV)[7]. The G band intensity of as-grown 12°-tBLG is around 45 times higher than that of monolayer graphene (MLG), and is 20 times higher than that of 2D peak of 12°-tBLG. The R peak, originating from the intervalley processes, is also nondispersive. The R peak of 12°-tBLG is centered at ~1500 cm^{-1}, and the peak position decreases with increasing twist angle[4]. For large twist angles, a D-like peak (centered at ~1350 cm^{-1}) and an R peak (centered in the 1370–1400 cm^{-1} range) can be observed (Fig. 1c, Supplementary Fig. 2b, c). Note that the D-like peak of tBLG is caused by the rotated interlayer stacking of the two layers, where one of the graphene layers would weakly perturb the other, rather than caused by in-plane disorder (point defects or grain boundaries)[4,8,9]. Nevertheless, to confirm this, Raman spectra were acquired in the monolayer region of the same domain of 27°-tBLG, which clearly shows the lack of the D band (Supplementary Fig. 2b).

Supplementary Note 3: Isotope-labelled growth of tBLGs for exploring the mechanism of hetero-site nucleation

To confirm the mechanism of our hetero-site nucleation strategy and capability of flow turbulence to initiate the nucleation of second layer, the $^{12}CH_4$ and $^{13}CH_4$ were switched every 5 min during the experiments. In detail, for the 30°-tBLG shown in Fig. 2c in the main text, the hetero-site nucleation began at the time point of 5 min by increasing the flow rates of H_2 (from 400 sccm to 1000 sccm) and CH_4 (from 0.4 sccm for $^{12}CH_4$ to 1.0 sccm for $^{13}CH_4$) (Fig. 2a, main text). For the ~9°-tBLG shown in Fig. 2d in the main text, the second layer was initiated at the time point of 10 min, when the carbon source was changed from $^{13}CH_4$ to $^{12}CH_4$ (Fig. 2b, main text). We can measure the grain sizes of each layer of tBLG at different times (5, 10, 15 and 20 min), and obtain the time evolution of grain size of the two graphene layers in a single domain, by analyzing the dosing sequence and 2D-band intensity Raman maps of ^{13}C and ^{12}C (Supplementary Fig. 4a, c). Our results imply that the grain sizes increase linearly with the growth time (Supplementary Fig. 4b, d), and therefore either top layer or second

layer grow at a constant rate. By extending the fitting line to make it intersect with the x axis (time axis), the obtained intercept is corresponding to the time when the nucleation of 2nd layer graphene occurred, and the nucleation time is consistent with the time when we increased the carbon source and partial pressure of H_2.

In contrast, the isotope-labelled growth of bilayer graphene without flow turbulence was carried out according to the sequence shown in Supplementary Fig. 5, which resulted in AB-stacked BLG (AB-BLG). These experiments confirmed as follows: 1) the nucleation of second graphene layer can be initiated by increasing H_2 and CH_4, irrespective of the $^{12}CH_4$ to $^{13}CH_4$ or $^{13}CH_4$ to $^{12}CH_4$ switching; 2) the nucleation site of the second layer can be controlled by the gas flow, which further highlights the importance of the hetero-site nucleation strategy.

Supplementary Note 4: Parameters for growing tBLGs

To determine the appropriate parameters for growing second-layer-free graphene (for first nucleation of the first layer) and second-layer-rich graphene (for hetero-site nucleation), gas flow windows (Fig. 3a and Supplementary Fig. 10) were plotted by conducting series of growth experiments without gas-flow perturbation. After annealing at 1020 °C under 500 sccm of H_2 for 30 min, growth proceeded under a flow of H_2 and CH_4. Besides the gap between the monolayer region and the bilayer region (Fig. 3a and Supplementary Fig. 10a), a clear gap is also observed between the bilayer region and the few-layer region in Supplementary Fig. 10b, which indicates that a higher partial pressure of H_2 produces thicker graphene. Therefore, we selected flow rates of 400 sccm and 0.4 sccm for H_2 and CH_4, respectively, for 5 min during the first nucleation step, after which the flow rate of H_2 was tuned from 400 sccm to 1500 sccm during the hetero-site nucleation step. To increase the probability of secondary nucleation at the edges of the first-layer islands, and to increase the carbon source supply for bilayer growth, we also increased the flow rate of CH_4 to 1.0 sccm and 1.6 sccm, respectively (0.4 sccm of CH_4 in the second step is the control group). Fig. 3b and Supplementary Fig. 10c, d suggest that appropriate H_2 flow ratio and CH_4 flow ratio are important. In this regard, values of the gas-flow parameters, namely $H_2 : CH_4$ = 400 sccm : 0.4 sccm during the first nucleation step, $H_2 : CH_4$ = 1000 sccm : 1 sccm during the hetero-site nucleation step, were selected for tBLG growth. Typical OM images of tBLGs are shown in Fig. 1b.

Supplementary Note 5: Characterizing the twist angle by using OM-method

Measuring the domain edges to estimate the twist angle is a rapid method for calculating the proportions of tBLG domains to all as-formed bilayer graphene domains. Since the H-terminated zigzag edge is the most stable edge considering our growth parameters[10,11], the sharp edges of graphene islands are widely used to roughly determine the crystallographic orientation[12-14]. Specifically, for the oblong-hexagonal islands, the long side is usually used to be defined as the zero-degree side[15]. Here we systematically compared the results derived from edge direction of domains and corresponding TEM SAED results of various graphene domains to improve accuracy of OM-based method. Consequently, 4 types of typical tBLG morphologies that we usually obtained and the rules for measuring the twist angles are summarized in Supplementary Table 1 and Supplementary Fig. 11.

Error is inevitable when measuring the orientations of each edge, because the limited resolution of OM image would contribute to the deviation of the edge recognition (Supplementary Fig. 12). Usually, the optical resolution σ is around 0.2 μm, which responds to the size of fuzzy region. Therefore, the error in determine one edge orientation is

$$\mathrm{Error}_{\varphi} = \arctan\frac{\sigma}{L} \quad \text{(S1)}$$

Where L is the length of domain edge, which usually range from ten micrometer to tens of micrometers. In this case, the Errorφ is calculated to be around 0.9°**.** Since the twist angle (θ) is determined by measuring the included angle between two edges of the two layers, the OM-based error of twist angle (**Error$_{\theta}$**) should be twice the value of **Errorφ**, which is about 2°.

Raman spectroscopy and corresponding maps are also employed to assist in the twist angle statistics. From the OM images (Supplementary Fig. 13a, Supplementary Fig. 14a) and Raman G-intensity (I_G) maps (Supplementary Fig. 13b, Supplementary Fig. 14b), the bilayer graphene can be easily observed, since I_G increases as the number of layers. The intensity of the 2D band (I_{2D}) and full width at half-maximum (FWHM) of the 2D band (Γ_{2D}) are used to check the stacking orders of the bilayer graphene. The bright regions in I_{2D} maps (Supplementary Fig. 9c, Supplementary Fig. 10c) indicate the tBLG domains, and AB-BLG domains show larger Γ_{2D} (50-60 cm^{-1}) (Supplementary Fig. 13d, Supplementary Fig. 14d). The I_{2D} for tBLG with small twist angle (<10°) is weaker than that for MLG

(Supplementary Fig. 14d). The R peak and R' peak are also characteristics for identifying tBLG domains (Supplementary Fig. 14e).

Therefore, by measuring the edges of the tBLG domains in OM images in conjunction with the Raman spectra, the ratio of tBLG and distribution of twist angles can be obtained (Supplementary Fig. 15). Considering the error in determining the twist angle by using the OM-based method, the bin width in the histogram is set to be 2° (Supplementary Fig. 15b and d).

Supplementary Note 6: Characterizing tBLG by TEM

Graphene samples were transferred onto commercially available TEM grids (Quantifoil) using a non-polymer-assisted method[16]. The structure of the TEM grid is shown in Supplementary Fig. 16, which consists of metal mesh and porous carbon film. We collected the diffraction patterns over all the intact graphene holes (suspended graphene on the carbon-film holes) for investigating the stacking order of BLG. We can obtain the proportions of tBLG and AB-BLG over all the bilayer graphene by analyzing the SAED patterns. Supplementary Fig. 17 shows the representative SAED patterns of MLG and AB-BLG. The intensity ratio of the diffraction points, $I_{\{2100\}}/I_{\{1100\}}$ in AB-BLG is clearly higher than that in MLG, which help us to distinguish AB-BLG from MLG by using the SAED patterns, despite each showing single groups of hexagonal points[6,17]. Therefore, the proportion of tBLG within BLG domains can be obtained by counting the numbers of tBLG and AB-BLG.

Supplementary Fig. 18 shows a library of SAED patterns of tBLG with the twist angle in the full range from 0° to 30°. The twist angle can be determined by measuring the direction of two opposite diffraction points of each layer graphene with much higher credibility than OM-based method. Since the diffraction points turn fuzzy when zoomed in, there still exits an inevitable error. Taking the ~2.6° SAED pattern as an example (Supplementary Fig. 19), the weight dashed lines we plotted along opposite diffraction points intersect each other with a twist angle. The error of determining the orientation of each SAED pattern (Errorφ) can be estimated by the full width at half maximum (FWHM) of the fuzzy diffraction points and the distance between each opposite bright point in single SAED pattern (bottom panel, Supplementary Fig. 19a).

$$\mathrm{Error}_{\varphi i} = \arctan\frac{FWHM_i}{2K_i} \tag{S2}$$

where i=1,2,3…, indicating the diffraction order, K_i is the distance from the center of the Brillouin zone to the diffraction point with the diffraction order of i. To further estimate the error of TEM-based method using different order of diffraction points, we plot the line profiles near the 1st, 2nd, 3rd, and 5th diffraction points (Supplementary Fig. 19b-e), where the FWHMs are all about 0.8 nm^{-1}. The vector of diffraction point K_i increases with the diffraction order i (K_1= 4.69 nm^{-1}, K_2= 8.12 nm^{-1}, K_3= 9.37 nm^{-1}, K_5= 14.06 nm^{-1}), indicating that the value $\mathbf{Error}_{\varphi}$ would be smaller if we choose higher order of diffraction points. Similar with the OM method, since we need measure the orientations of each layer, the error of twist angle ($\mathbf{Error}_{\theta}$) should be two folds of the $\mathbf{Error}_{\varphi}$, and the obtained $\mathbf{Error}_{\theta}$ using the points of different diffraction orders are shown in Supplementary Table 2.

The camera length (L_{camera}) and aperture size are also important parameters related to the error when determining the crystal orientation by using the SAED method. The distance from the diffraction point to the diffraction center on the screen (R) is proportional to L_{camera}: $R = L_{camera}\lambda/d$, where λ is the wavelength of the electron beam, and d is the lattice of graphene. Therefore, a larger the camera length would allow us obtain a larger R on screen and therefore a smaller error. The aperture size would influence the intensity of the diffraction points. Therefore, we investigate these factors by conducting a series experiment by varying the camera length (750 mm, 560 nm and 340 mm) and aperture size (diameter of 200 nm and 800 nm). The corresponding results are listed in Supplementary Table 3, and the $\mathbf{Error}_{\theta}$ measured by using the 1st and 2nd order diffraction points can reach 0.44° with camera length 750 mm or 560 mm.

Supplementary Note 7: Characterizing tBLG by ARPES

Supplementary Fig. 25 shows the micro-ARPES data collected from three obtained CVD-grown tBLG domains on Cu substrate. By overlapping the Brillouin zones of graphene layers with ARPES intensity maps, the twist angle θ of ~3°, ~6°, and ~11° (±1°) can be determined, respectively (Supplementary Fig. 25a-c). We also plotted the schematic diagrams of band structure for these tBLGs near the Dirac cones to show the twist-angle dependent band structure (inset of Supplementary Fig. 25a-c). The vHSs arising from the interlayer coupling in band-crossing area are clearly observed in the energy-momentum-dispersion (the left panel of Supplementary Fig. 25d-f), where the top layer displays a relatively higher intensity in each figure. The corresponding integrated energy distribution curves (EDCs) are also plotted (right panel of Supplementary Fig. 25d-f) to quantitatively determine

the position of vHSs. Considering the substrate doping effect on the tBLGs, the ΔE_{vHS} can be calculated by the formula $\Delta E_{vHS} = 2|E_D - E_{vHS}|$, where E_D is the energy of Dirac point. Clearly, as the θ increases, the ΔE_{vHS} increases as well, consistent with previous reports[18]. Our observation confirms that the electronic structure of our tBLGs is twist-angle-dependent, which endows tBLGs the θ-dependent-enhanced optical absorption, θ-dependent-enhanced Raman G-band intensity and enhanced photocurrent generation at certain wavelengths[7,19,20].

Supplementary Note 8: Discussion on the formation of interlayer twist

Three kinds of interactions are important in determining the orientation of graphene: 1) the interaction between graphene edge and Cu atoms (G_{edge}-Cu); 2) the interaction between the plane of graphene to Cu substrate (G_{plane}-Cu); 3) the interlayer interaction between graphene plane to graphene plane (G_{plane}-G_{plane}). The schematic diagrams and corresponding energies of these interactions are shown in Supplementary Fig. 26a-c, which reveal that interaction of G_{edge}-Cu is clearly stronger than that of G_{plane}-G_{plane}. To determine the dominant orientation of a graphene nucleus, the fluctuation of the formation energy, ΔE of tBLGs with different orientations is important. Taking C_{54} and C_{1014} (the size of C_{1014} is ~5.6 nm) as examples, ΔE (G_{edge}-Cu of C_{54}) = 5.18 eV and ΔE (G_{edge}-Cu of C_{1014}) ≈ 100 eV, respectively[1]. However, the energy difference between AB stacking bilayer graphene and twisted bilayer graphene ($\Delta E_{AB\text{-}twist}$) is only about 0.216 eV and 4.056 eV for C_{54} and C_{1014}, respectively[21]. Clearly, $\Delta E_{AB\text{-}twist}$ is lower than ΔE(G_{edge}-Cu) for carbon clusters, indicating that the interaction between Cu edge atoms and graphene edge atom would be mainly responsible for determine the orientation of second layer graphene. Therefore, the interaction from the Cu substrate would suppress the equilibrium AB-stacking configuration to form interlayer twist.

In another hand, there exist steps, kinks, dislocations and particles on the Cu surface, which could serve as nucleation sites for bilayer graphene. These the various atomic arrangement of the Cu step edge ensure the capability to growth bilayer graphene with a wide range of orientation. For the step-attached nucleation along $(01\bar{1})$ direction on Cu(111) surface, two orientations (0° and 30°) are energetically favorable (Supplementary Fig. 26d)[2]. For high-index facets, the atomic arrangements of edge atoms turn more complex[22], which is key for forming a wide range of interlayer twist (Supplementary Fig. 26e). Admittedly, the in-depth mechanism is still very unclear at the moment, we believe the kinks on high-index Cu surface with various orientation is important for suppressing

the AB-stacked equilibrium. The nucleation near the particles on pre-melting Cu substrate is also very common, because of the reduced nucleation barrier. These particles would also enable the formation of second layer graphene with rich twist angles, because of Euclidean geometry of particles[23]. In addition, the particles on Cu surface is commonly observed, and would move on the Cu surface during the growth of graphene[24]. Therefore, if the nucleation site of the secondary layer is near the particles, and at different site from that of the first layer, their surrounding environments would be totally different (Supplementary Fig. 26d-f).

Supplementary Tables

Supplementary Table 1. Rules for measuring the twist angles.

Type	Description	Rules	Schematic diagrams
I	Regular hexagon<br>Oblong-hexagon, internal angles of which are still all 120°	Take any two corresponding edges of each layer (dashed blue and red line), and measure the included angle between two lines	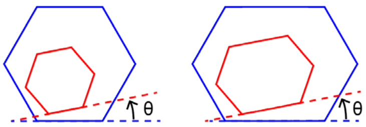
II	1. Oblong-hexagonal shape<br>2. All opposite edges of the hexagon are parallel but the angle between the long side and the adjacent side is **not** 120°	Take the longest sides as referring lines, and measure the included angle between two longest lines	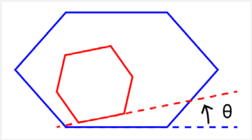
III	1. Oblong-hexagonal shape<br>2. One or two pair of opposite edges of the hexagon are **not** parallel<br>3. The long edge of the hexagon is parallel to the opposite edge	Take the long sides as referring lines, and then measure the included angle between two longest lines	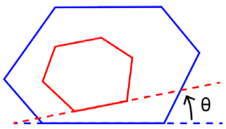
IV	1. Oblong-hexagonal shape<br>2. The long edge of the hexagon is not parallel to the opposite edge<br>1. Oblong-hexagonal shape<br>2. None of the pair of opposite sides are parallel	Take the long sides as referring lines, and then measure the included angle between two longest lines, but the error is larger than type III	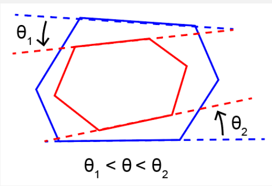

Supplementary Table 2. Errors of SAED method by measuring the tilt angles of diffraction points with different diffraction orders

Selected diffraction order (i)	1	2	3	5
K_i (nm^{-1})	4.69	8.12	9.37	14.06
Tilt angle error: $Error_\varphi$ (°)	0.49	0.28	0.24	0.16
Twist angle error: $Error_\theta$ (°)	0.98	0.56	0.48	0.32

Supplementary Table 3. Errors of SAED method by measuring the tilt angles of diffraction points with different diffraction orders

Aperture (d, nm)	Camera length (mm)	diffraction order (*i*)	K_i (nm^{-1})	Tilt angle error: $Error_\varphi$ (°)	Twist angle error: $Error_\theta$ (°)
200	750	1	4.69	0.22	0.44
		2	8.12	0.22	0.44
	560	1	4.69	0.34	0.68
		2	8.12	0.22	0.44
	340	1	4.69	0.32	0.64
		2	8.12	0.27	0.54
800	750	1	4.69	0.23	0.46
		2	8.12	0.22	0.44
	560	1	4.69	0.30	0.60
		2	8.12	0.24	0.48
	340	1	4.69	0.31	0.62
		2	8.12	0.27	0.54

Supplementary Figures

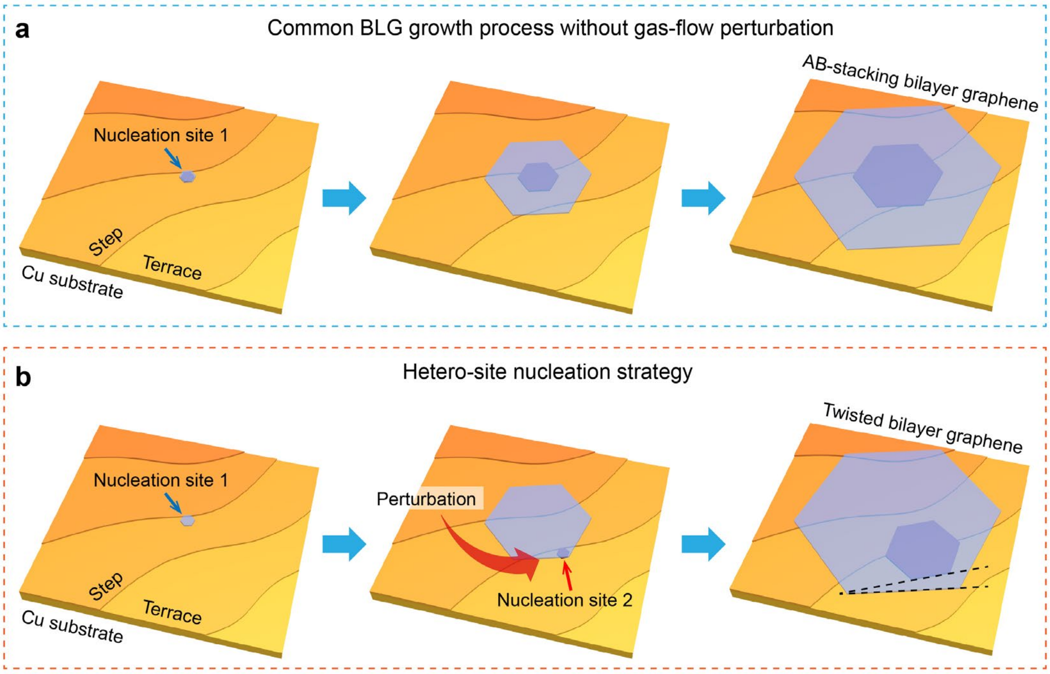

Supplementary Fig. 1: Schematic diagrams of common growth process and hetero-site nucleation strategies for growing (a) AB-BLG and (b) tBLG.

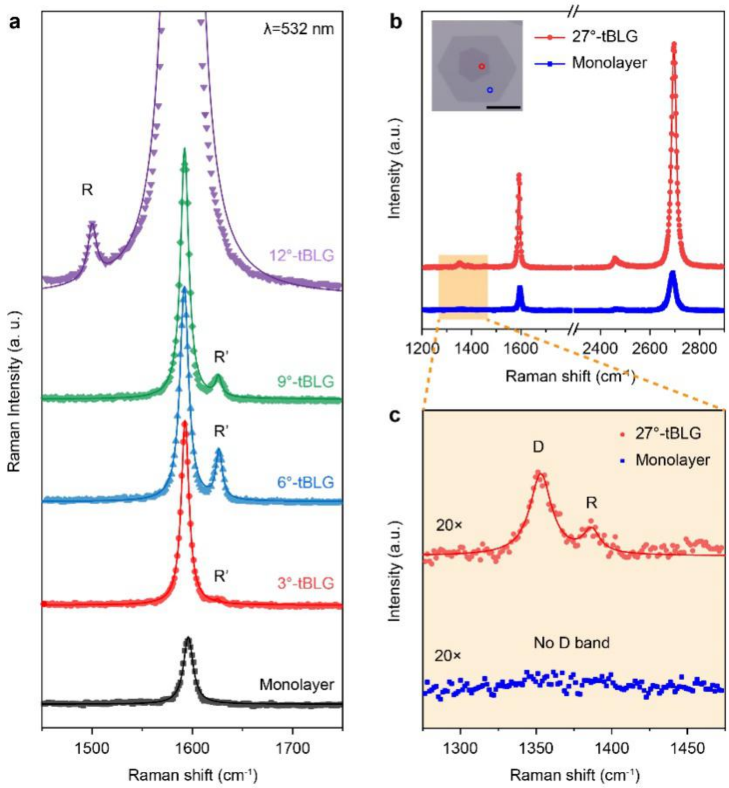

Supplementary Fig. 2: Raman spectra of tBLG. a, Raman spectra of 3°-tBLG, 6°-tBLG, 9°-tBLG and 12°-tBLG in the frequency range from 1450 to 1750 cm^{-1}. **b,** Raman spectra of the monolayer graphene and 27°-tBLG regions in the same domain. Inset: OM image of 27°-tBLG; scale bar: 10 μm. **c,** Enlarged Raman spectra in panel (**b**) in the frequency range from 1275 to 1475 cm^{-1}.

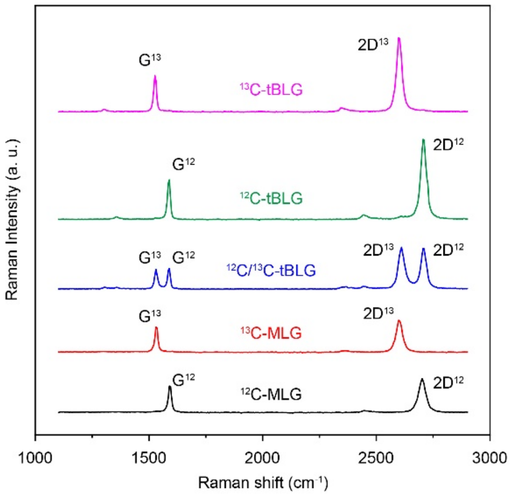

Supplementary Fig. 3: Raman spectra of ^{12}C monolayer graphene (^{12}C-MLG, black), ^{13}C monolayer graphene (^{13}C-MLG, red), ^{12}C/^{13}C twisted bilayer graphene (^{12}C/^{13}C-tBLG, blue), ^{12}C twisted bilayer graphene (^{12}C-tBLG, green), and ^{13}C twisted bilayer graphene (^{13}C-tBLG, purple). Note that the twist angle is about 30°.

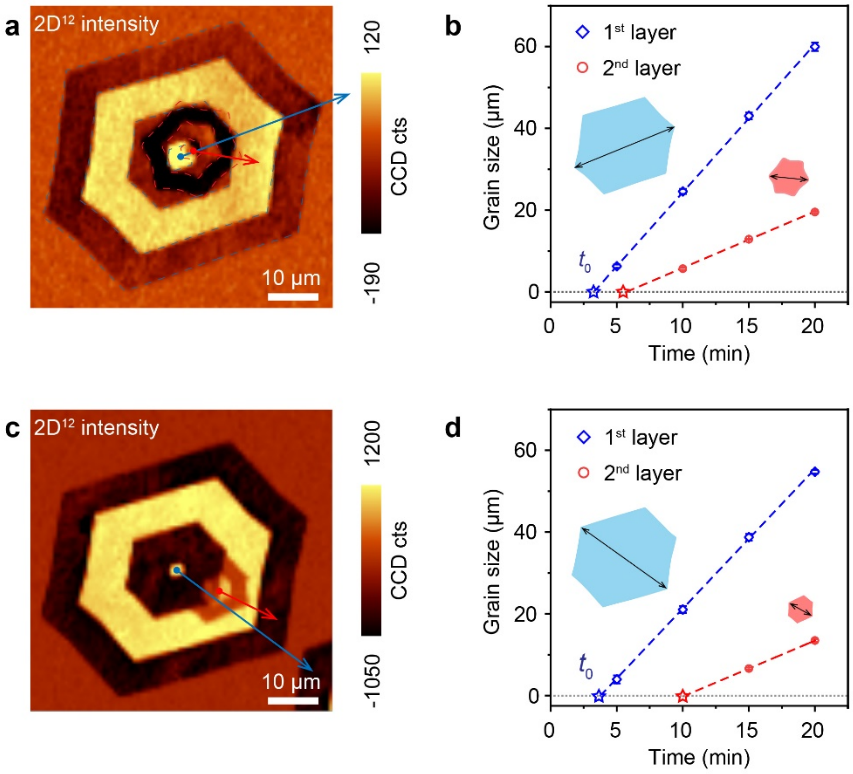

Supplementary Fig. 4: Raman 2D^{12}-intensity maps and corresponding schematic diagrams depicting the growth of isotope-labelled tBLGs. a,c, Raman 2D^{12}-intensity maps (integrated from 2660 cm^{-1} to 2720 cm^{-1}), which complement the 2D^{13}-intensity maps (integrated from 2560 cm^{-1} to 2620 cm^{-1}) shown in Fig. 3e, f in the main text. **b,d,** The grain sizes (diagonal) of the 1st-layer (blue) and the 2nd-layer (red) graphene as functions of growth time corresponding to (**a**) and (**c**), respectively.

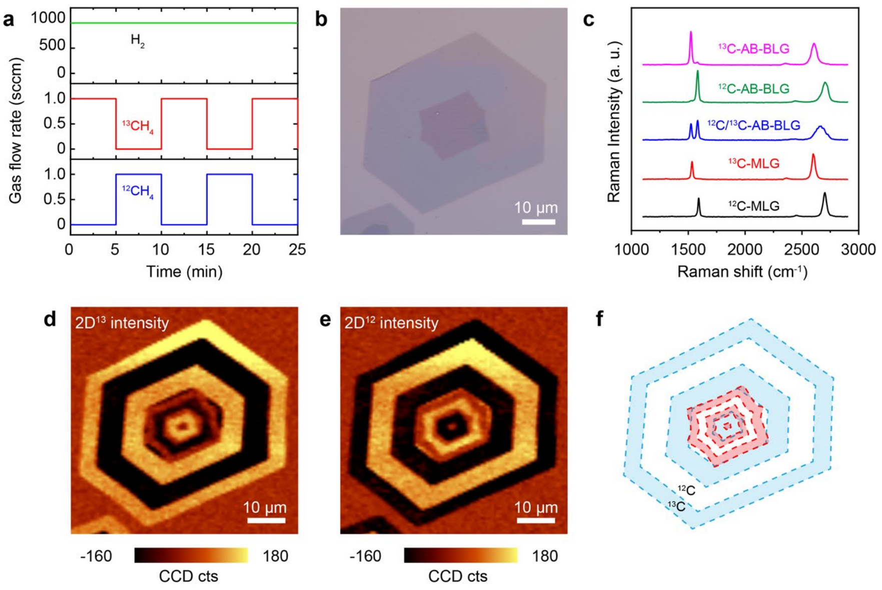

Supplementary Fig. 5: Isotope-labelled growth of AB-BLG using the normal one-step method. **a,** Feedstock feeding process for AB-BLG-growth. **b,** OM image of as-grown AB-BLG on a SiO_2/Si substrate. **c,** Raman spectra of ^{13}C-AB-BLG (purple), ^{12}C-AB-BLG (green), ^{12}C/^{13}C-AB-BLG (blue), ^{13}C monolayer graphene (red), and ^{12}C monolayer graphene (black). **d,e,** Raman 2D^{12}-intensity (**d**, integrated from 2660 cm^{-1} to 2720 cm^{-1}) and 2D^{13}-intensity (**e**, integrated from 2560 cm^{-1} to 2620 cm^{-1}) maps of the AB-BLG corresponding to panel (**b**). **f,** Schematic diagram of the AB-BLG growth process corresponding to (**d, e**). Scale bars: 10 μm.

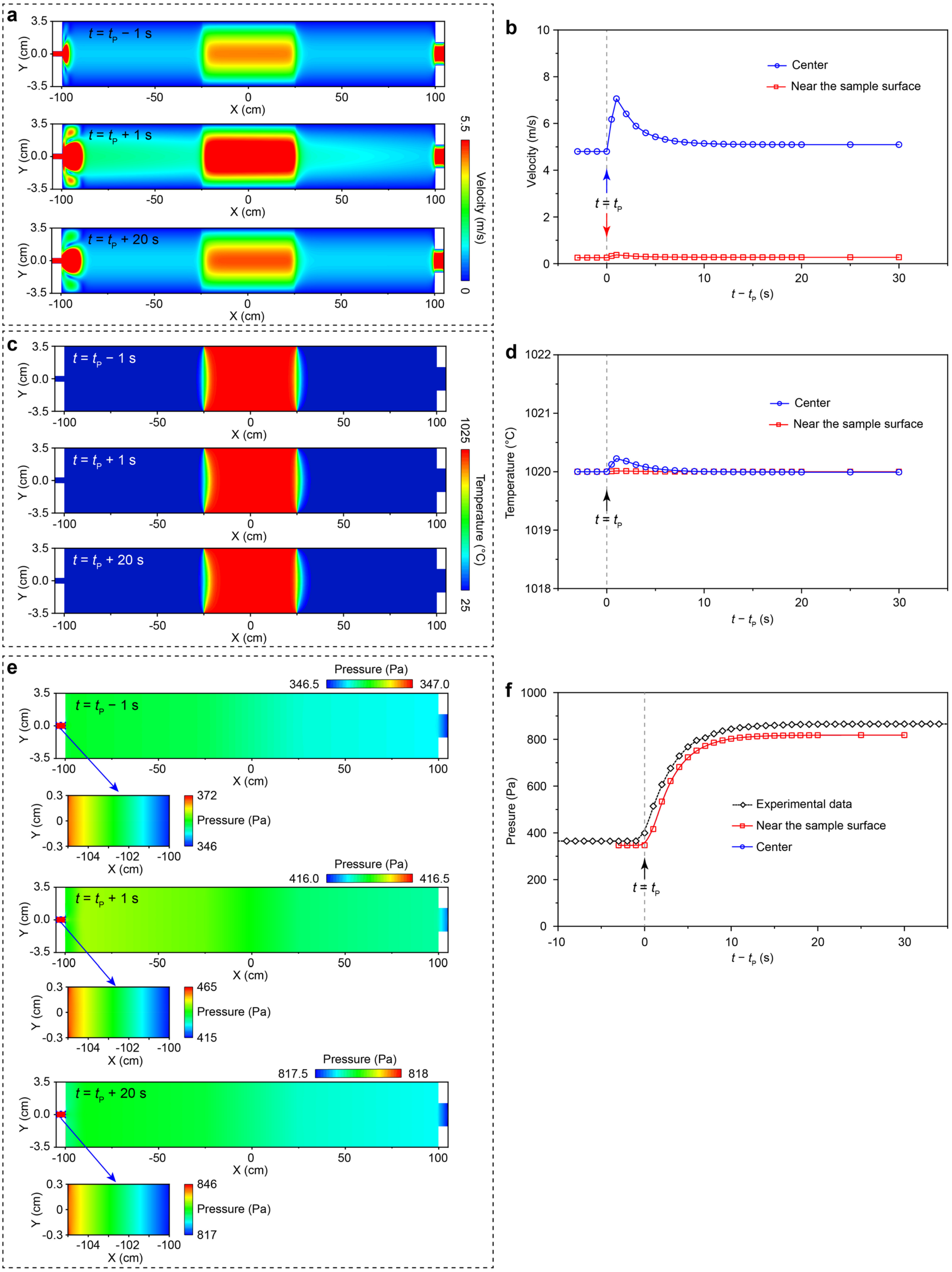

Supplementary Fig. 6: Computational fluid dynamics (CFD) simulation results on the dynamics of gas-flow velocity, temperature and pressure during the gas-flow perturbation. a, Spatial distribution of velocity in the tube reactor at $t = t_p - 1$ s, $t = t_p + 1$ s, and $t = t_p + 20$ s, respectively. **b,** The gas-flow velocity at the center of the tube and the velocity near the sample surface (~ 1 mm) as

a function of time during the perturbation. **c,** Spatial distribution of temperature in the tube reactor at $t = t_P - 1$ s, $t = t_P + 1$ s, and $t = t_P + 20$ s, respectively. **d**, Temperature as a function of time during the perturbation. Only a small rise of temperature (0.3°C) at the center of the tube and almost no temperature fluctuation near the sample surface (~1 mm from the sample surface) are observed, which would have negligible influence on the CVD graphene growth. **e**, Spatial distribution of pressure in the tube reactor at $t = t_P - 1$ s, $t = t_P + 1$ s, and $t = t_P + 20$ s, respectively. **d**, Simulated results of pressure (at the center of the tube and near the sample surface) and experimentally measured pressure as a function of time during the perturbation. Note that the real-time pressure would reach 80% of the target value within 5 seconds after the perturbation. t_P: the time when the perturbation was introduced.

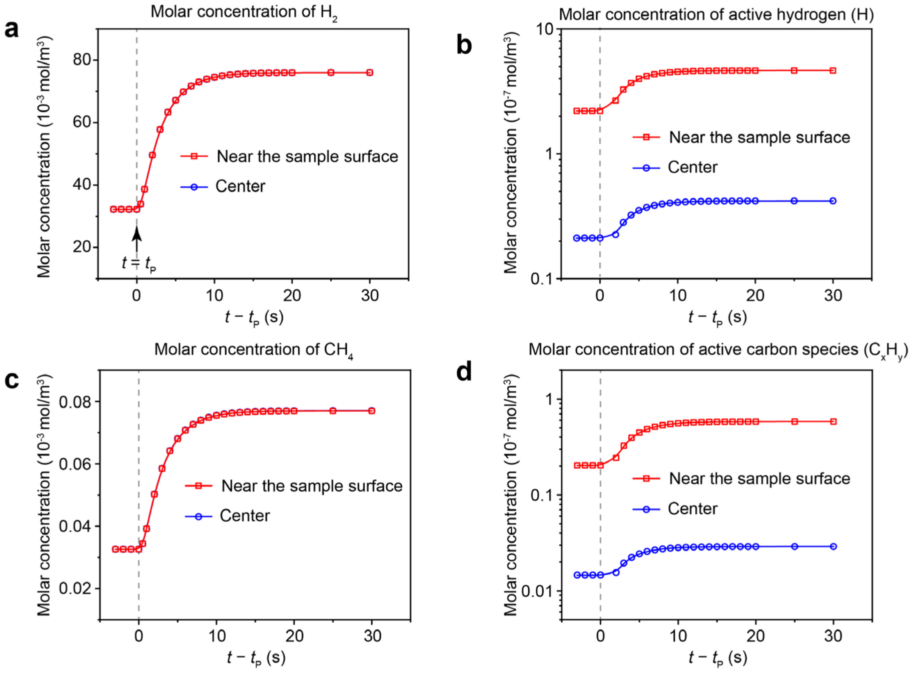

Supplementary Fig. 7: CFD simulation results on the dynamics of gas mixture/concentration. a,b Molar concentration of H_2 (a) and active hydrogen H (d) as functions of time. c,d, Molar concentration of CH_4 (c) and active carbon species (C_xH_y) (d) as functions of time. t_P: the time when the perturbation was introduced.

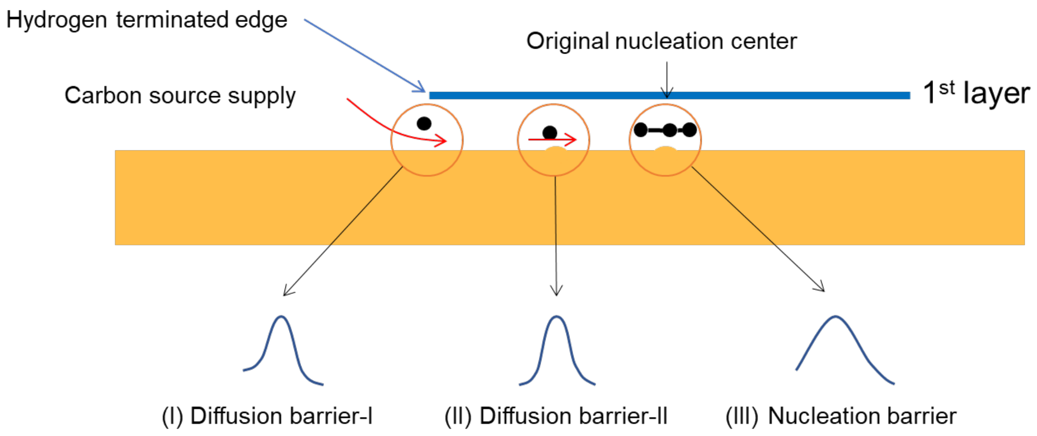

Supplementary Fig. 8: Energy barriers that the carbon species must overcome to forming the second-layer nuclei near the center of the first-layer.

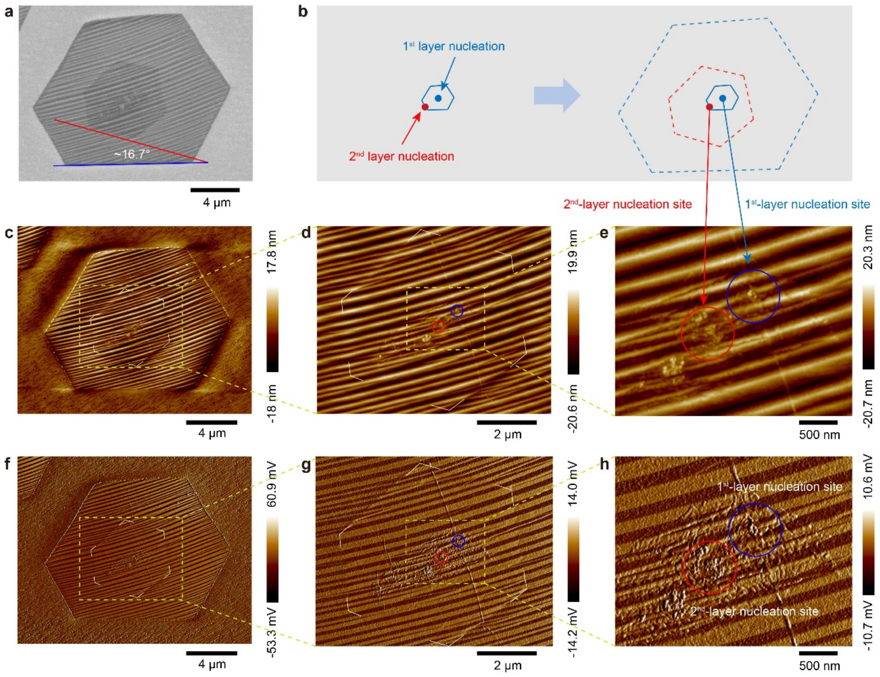

Supplementary Fig. 9: One example of substrate effect on the hetero-site nucleation of the second layer graphene. (a) SEM image of a tBLG. (b) Schematic diagram displaying the nucleation site of the second layer. (c-e) AFM height images zoomed in the region near the nucleation center. (f-h) AFM peak force error images corresponding to (c-e), which show clearer edges of the tBLG.

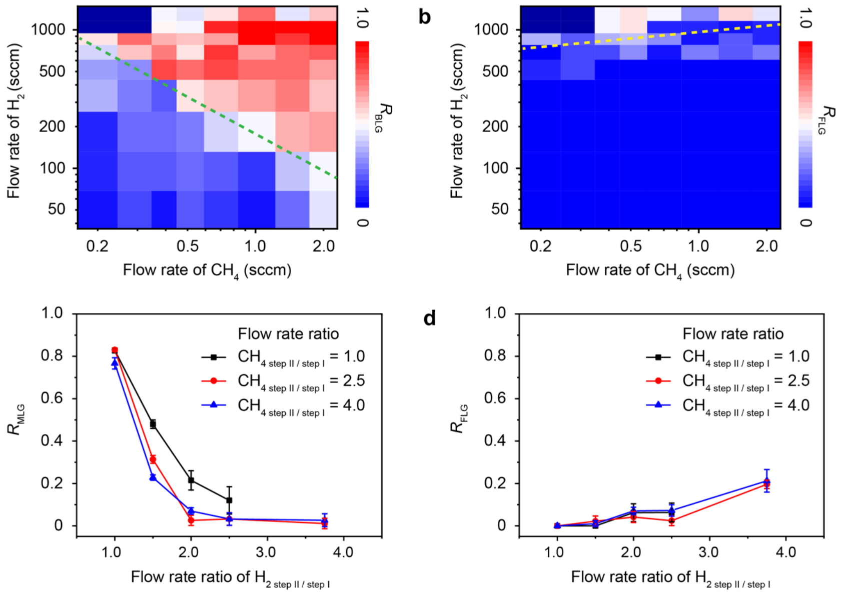

Supplementary Fig. 10: Gas flow parameters for growing bilayer and few-layer graphene with and without using hetero-site nucleation strategy. a,b, Ratios of (**a**) bilayer graphene and (**b**) few-layer graphene (FLG) domains as functions of H_2 and CH_4 flow rates. **c,d,** Ratio of (**c**) monolayer graphene (MLG) and (**d**) FLG domains as functions of the ratio between the H_2 flow rate during hetero-site nucleation step and that during the first nucleation step. Black, red and blue curves indicate that the ratio between the flow rate of CH_4 during hetero-site nucleation step and that during the first nucleation step are 1.0, 2.5, and 4.0, respectively.

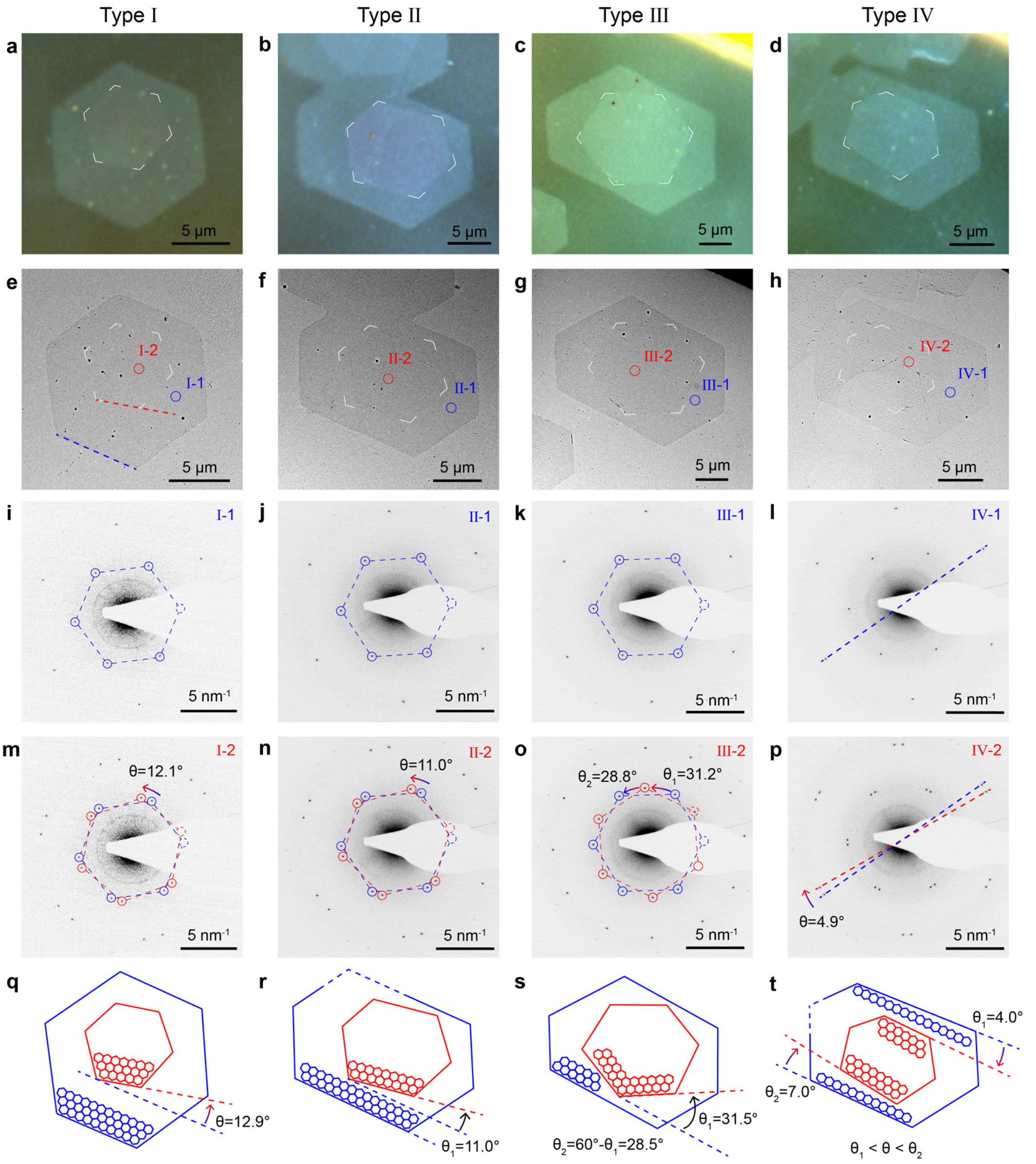

Supplementary Fig. 11: Rules for determining the twist angles based on OM images. (a-d) OM images of 4 typical types of tBLGs on coordinated TEM grid. (e-h) TEM images of the 4 types of tBLGs corresponding to (a-d). (i-l) SAED pattern of monolayer region taken from the region marked with blue circles in (e-h). (m-p) SAED patterns of tBLG taken from the regions marked with red circles in (e-h). (q-t) schematic diagram of measuring the twist angle, where the dashed lines indicate the edges of graphene, and the zigzag edges of honeycomb structure are derived from the SAED patterns according to the Fourier transform relationship.

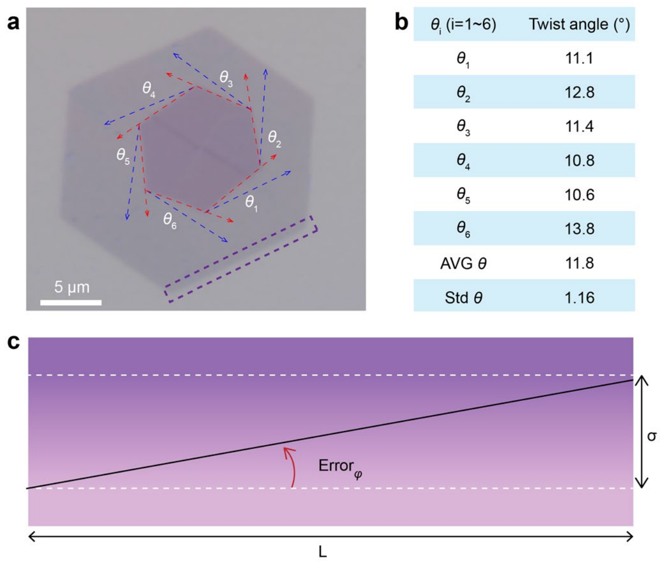

θ_i (i=1~6)	Twist angle (°)
θ_1	11.1
θ_2	12.8
θ_3	11.4
θ_4	10.8
θ_5	10.6
θ_6	13.8
AVG θ	11.8
Std θ	1.16

Supplementary Fig. 12: Error analysis on the twist-angle measurement of tBLG on SiO_2 by OM method. (a) A typical OM image of tBLG on SiO_2. (b) Twist angles measured between 6 pairs of hexagonal edges of tBLG. (c) Illustration of the origin of the one-side direction error during the measurement.

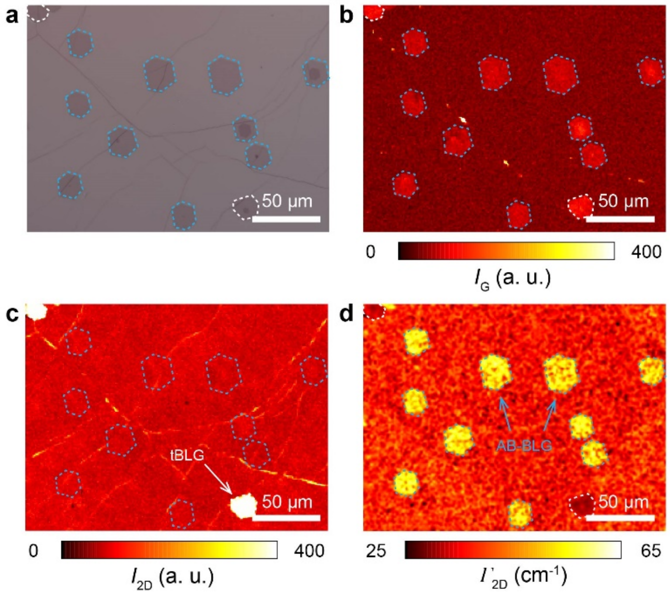

Supplementary Fig. 13: Typical OM image and Raman mapping of bilayer graphene without hetero-site nucleation. a, OM image of BLGs. The dashed lines help to confirm AB-BLGs (blue) and tBLGs (white). **b-d,** Raman mapping showing intensity of G band (**b**), intensity of 2D band (**c**), and width of 2D band (**d**), for the selected area in (**a**).

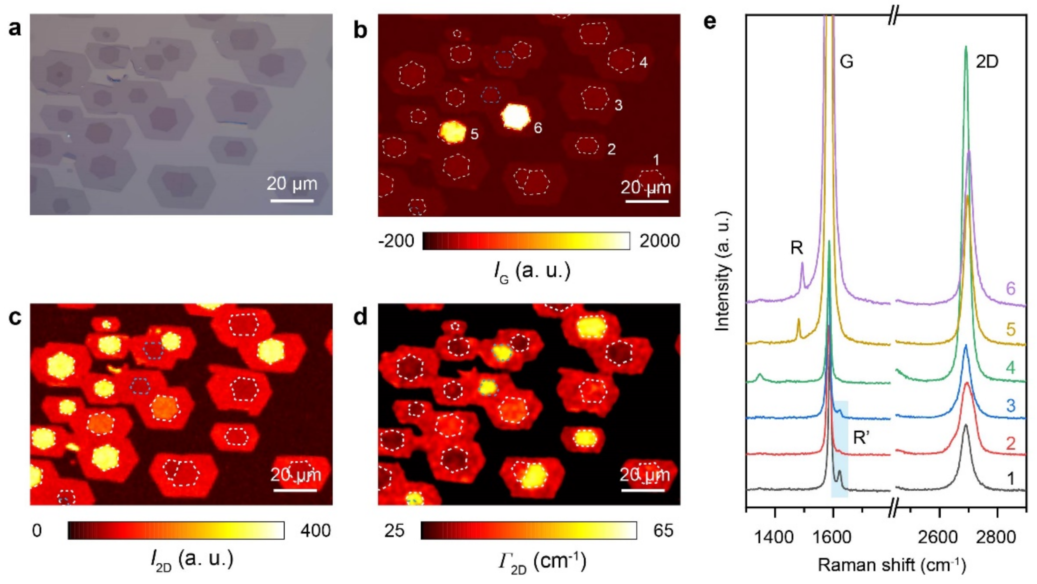

Supplementary Fig. 14: Typical OM image and Raman mapping of bilayer graphene with hetero-site nucleation. a, Optical microscopy image of BLGs. **b-d,** Raman mapping showing intensity of G band (**b**), intensity of 2D band (**c**), and width of 2D band (**d**), for the selected area in (**a**). The dashed lines help to confirm BLG domains. **e**, Raman spectra of tBLGs corresponding to the samples marked in panel (**b**).

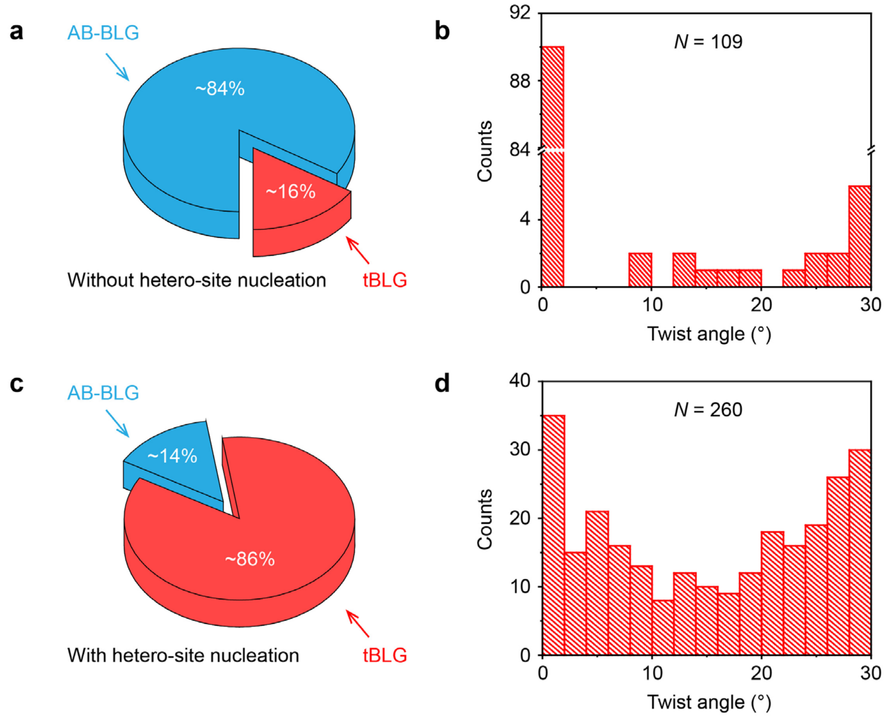

Supplementary Fig. 15: Ratio of tBLG and AB-BLG and distribution of twist angles in as-grown tBLGs obtained by measuring the edges of hexagonal graphene domains using OM results. (a,b) Pie charts of stacking order (AB-BLG or tBLG) for graphene growth without hetero-site nucleation strategy (a) and the corresponding distribution of twist angles (b). (c,d) Pie charts of stacking order (AB-BLG or tBLG) for graphene growth with *hetero-site* nucleation strategy (a) and the corresponding distribution of twist angles (b).

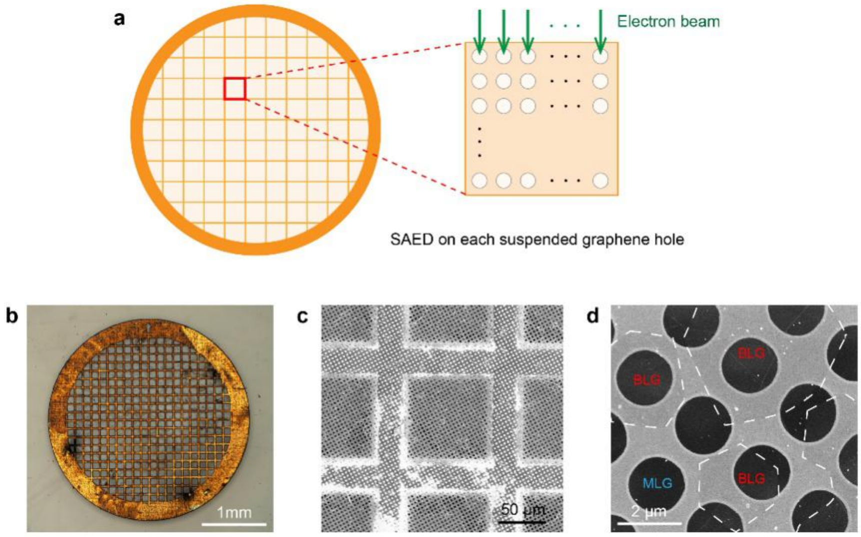

Supplementary Fig. 16: Preparing a sample of as-grown tBLGs for TEM. a, Schematic of the TEM grid and the process for collecting SAED results. **b,** OM image of the TEM grid covered by the tBLG sample. **c,d** SEM images of the as-transferred tBLG sample on the TEM grid. MLG and tBLG are distinguishable by contrast.

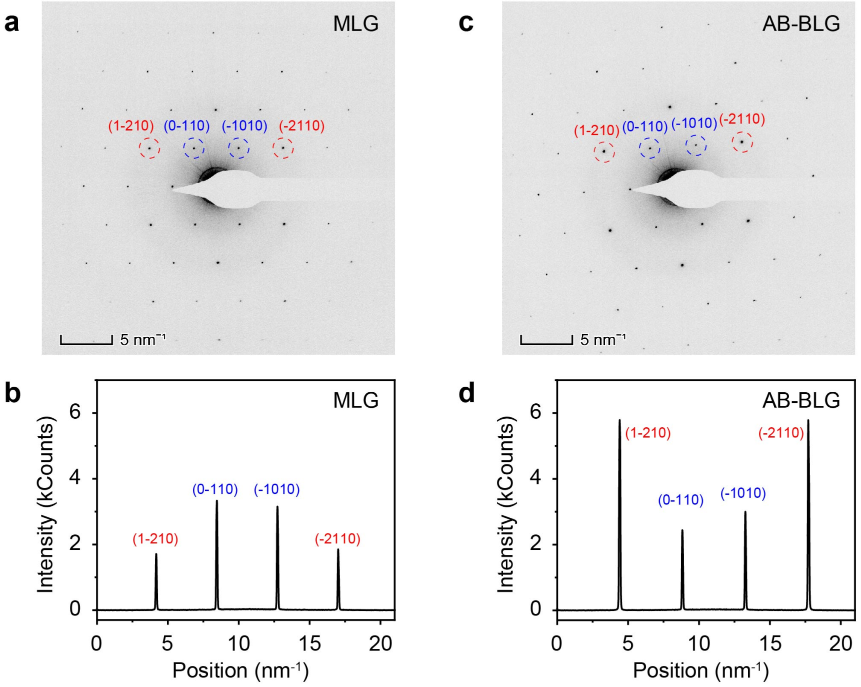

Supplementary Fig. 17: Typical diffraction patterns and corresponding spot intensity profiles of MLG (a, b) and AB-BLG (c, d).

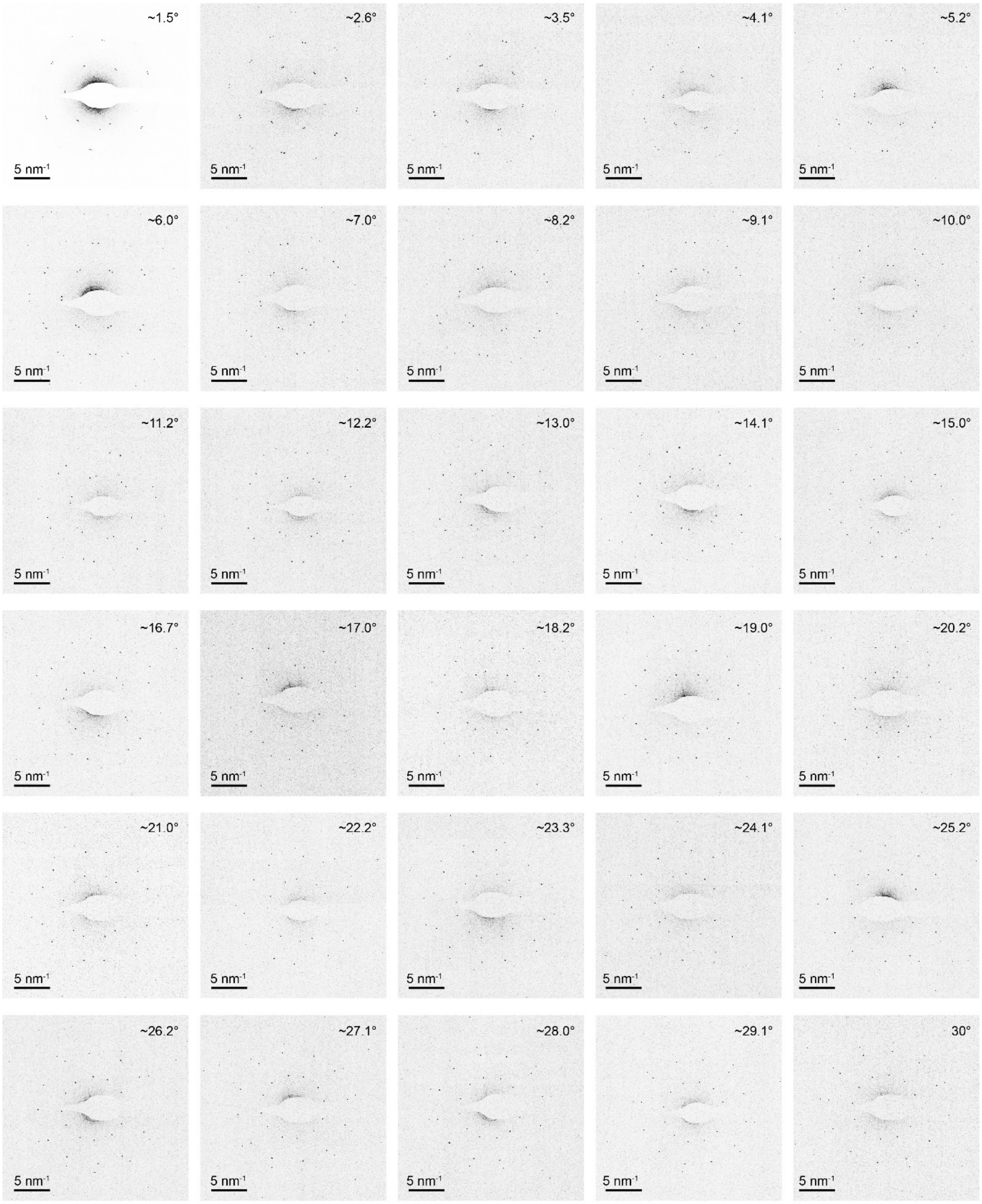

Supplementary Fig. 18: Library of SAED patterns in the full 0–30° range of interlayer twist angles.

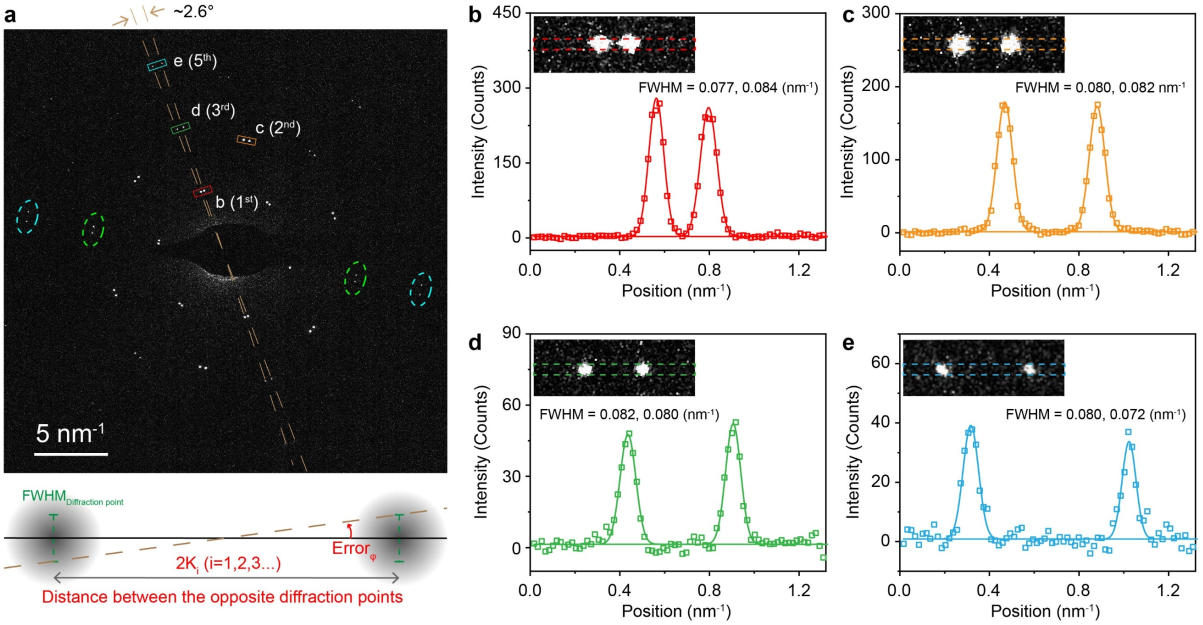

Supplementary Fig. 19: Error analysis on the twist-angle measurement of the tBLG domain by TEM (SAED) method. (a) A typical SAED pattern (top) and the illustration on the error of the tilt angle determined by one sets of diffraction points. (b-e) Diffraction intensity profiles versus position of four diffraction points shown in (a), where FWHMs are marked.

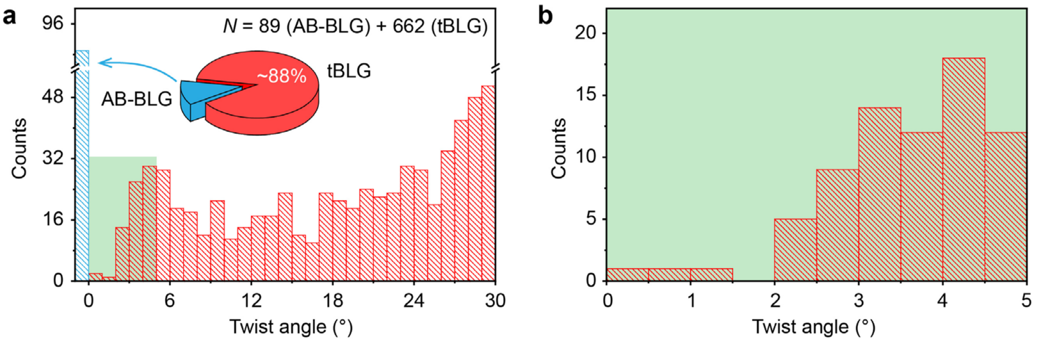

Supplementary Fig. 20: Statistical results of twist angles. (a) Statistical results of stacking order (AB stacking or non-AB stacking) and distribution of twist angles based on SAED patterns of as-grown BLGs. (b) A second histogram which is magnified to cover only the region below 5° twist angle, with the bin width of 0.5°. Here, the hetero-site nucleation strategy, in which different local environments plays crucial roles in determining the orientations of two graphene layers, enables the formation of small twist angles (< 3°).

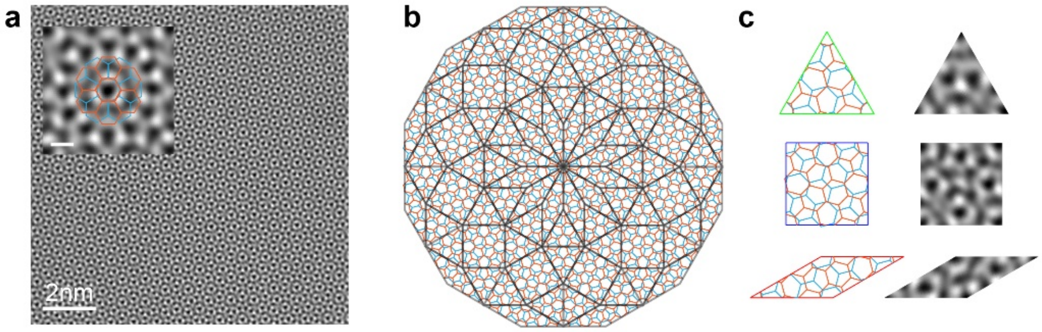

Supplementary Fig. 21. HR-TEM image and crystal structure of 30°-tBLG quasicrystal. a, HR-TEM image of 30°-tBLG. Inset: enlarged lattice image with corresponding crystal structure; Scale bar: 0.2 nm. **b,** dodecagonal quasicrystal structure of 30°-tBLG. **c,** Structrue units of dodecagonal quasicrystal and corresponding HR-TEM images.

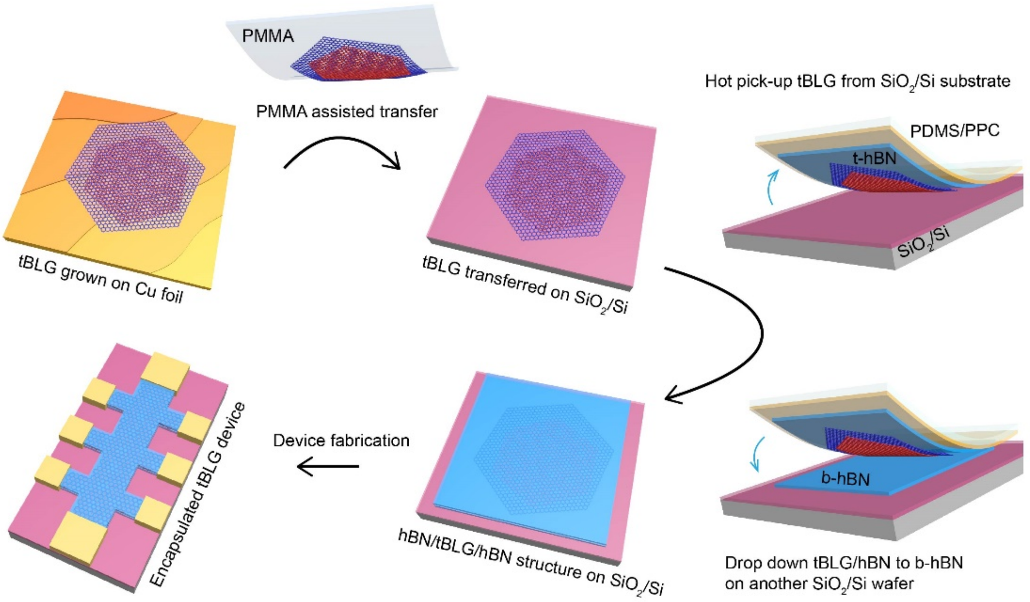

Supplementary Fig. 22: Schematic for the fabrication of the hBN-encapsulated tBLG Hall bar device with one-dimensional edge contact.

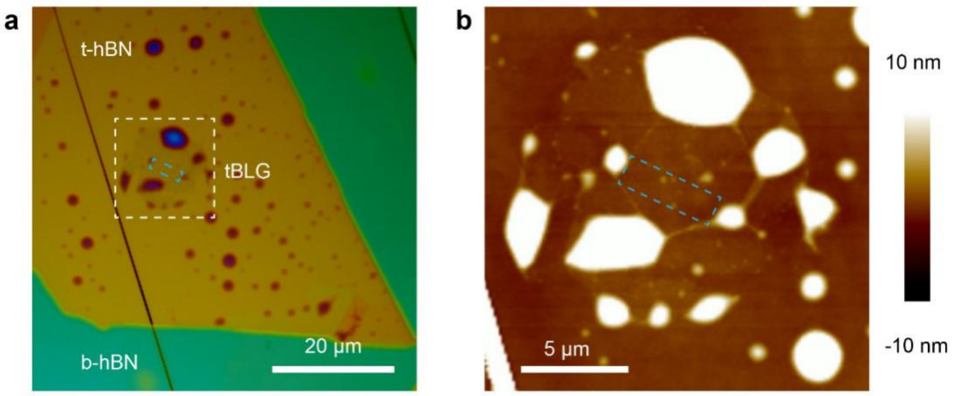

Supplementary Fig. 23: OM and AFM images of selected area of Hall bar device. a, Enhanced OM image of as-fabricated hBN/tBLG/hBN structure. **b,** AFM image corresponding to the region in the dashed white box in (**a**). The region enclosed in the dashed green rectangular was selected to fabricate the Hall bar device.

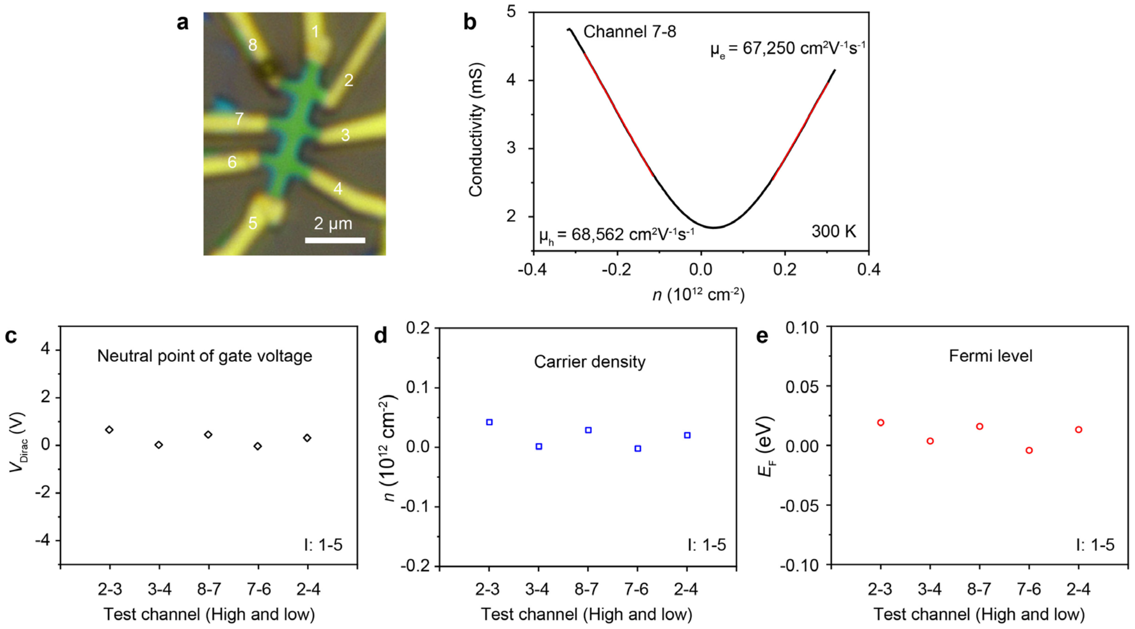

Supplementary Fig. 24: Carrier mobility and Doping-level of as-grown tBLG in the Hall bar device. a, OM image of the Hall bar device of encapsulated tBLG. **b,** Linear fit of the conductivity σ (channel 7-8) as a function of the charge-carrier density (n). (c-e) Extracted neutral point of gate voltage (V_G), carrier density (n), and Fermi level (E_F).

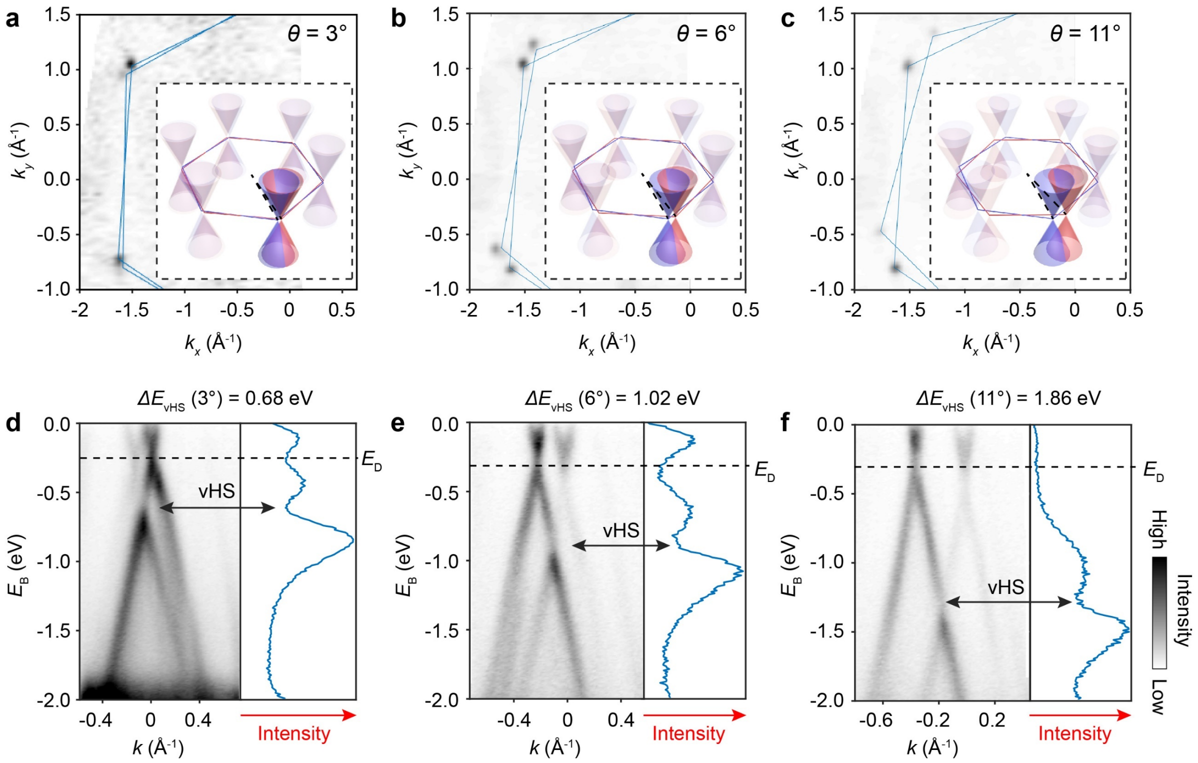

Supplementary Fig. 25: Electronic band structure of tBLGs with various twist angles. Micro-ARPES data collected from three obtained CVD-grown tBLG domains on Cu substrate. (a-c) ARPES equal energy contours for the tBLGs with the twist angle of ~3° (a), ~6° (b), and ~11° (c), respectively. Inset: schematic diagrams of the reciprocal space BZs and two sets of Dirac cones of tBLGs. (d-f) Energy-momentum-dispersion along $K - K_\theta$ (left panel) with the twist angle of ~3° (d), ~6° (e), and ~11° (f), respectively. The corresponding integrated energy distribution curves (EDCs) are presented in the right panel, where the vHSs are indicated. The dashed blank lines indicate the energy of the Dirac point (E_D).

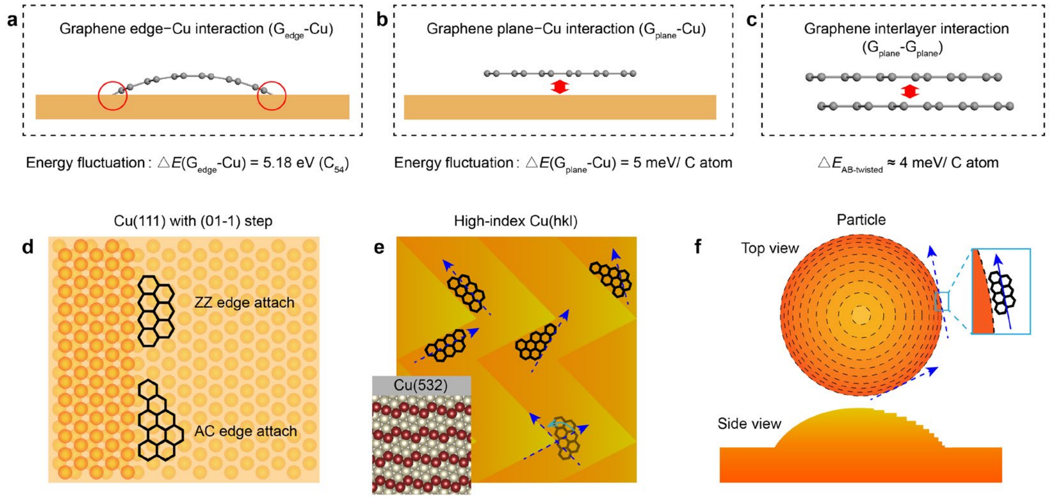

Supplementary Fig. 26: Roles of Cu substrate in determining the orientation of graphene nuclei. (a-c) Illustration of three kinds of interactions and related energies, which including graphene edge-Cu interaction (a), graphene plane-Cu interaction (b), and graphene interlayer interaction (c)[1,21]. (d-f) Possible structures of graphene nuclei on various Cu surface with steps (d), kinks (e), and particles (f).

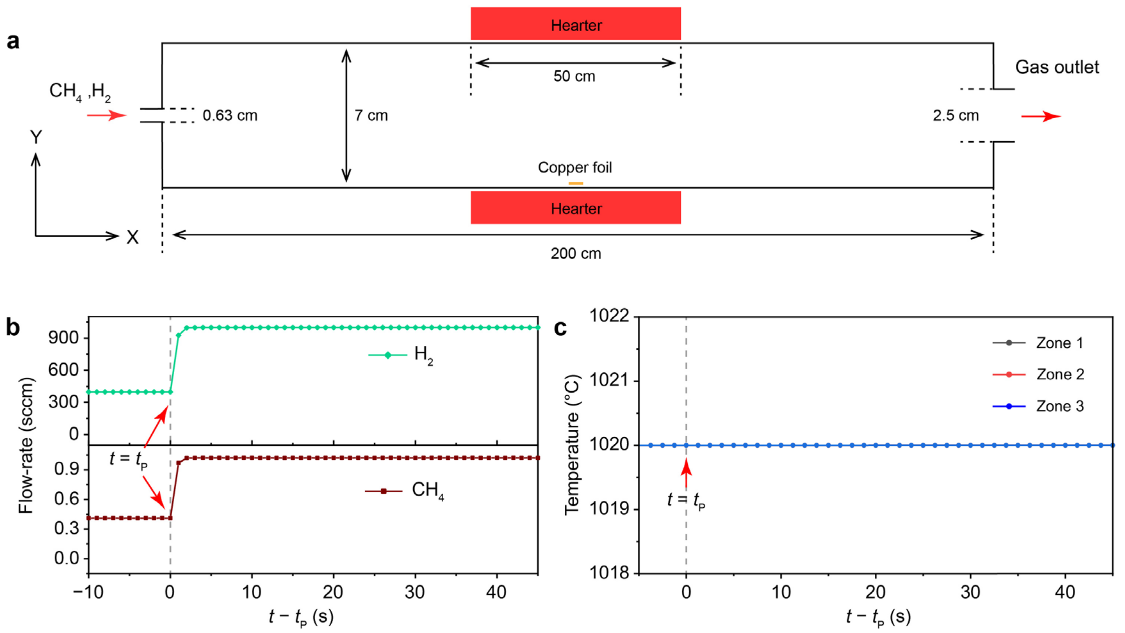

Supplementary Fig. 27: Model and boundary conditions of our CFD simulation. a, Geometry of the tube reactor. **b**, Gas flow settings in our simulation, which is based on the measurement results from the flowmeters we used (HORIBA METRON, S48 32/HMT) during the perturbation. **c**, Measured temperatures obtained from three thermocouples (three hot zones) near the wall of tube reactor we used during the gas-flow perturbation. The outlet boundary condition is based on the pump performance curve, which is obtain from the specification of our mechanical pump (ULVAC, GCD-136X). t_P: the time when the perturbation was introduced.

Supplementary References:

1. Zhang, X. Y., Xu, Z. W., Hui, L., Xin, J. & Ding, F. How the Orientation of Graphene Is Determined during Chemical Vapor Deposition Growth. *J. Phys. Chem. Lett.* **3**, 2822-2827 (2012).
2. Yuan, Q. H., Yakobson, B. I. & Ding, F. Edge-Catalyst Wetting and Orientation Control of Graphene Growth by Chemical Vapor Deposition Growth. *J. Phys. Chem. Lett.* **5**, 3093-3099 (2014).
3. Yan, Z. *et al.* Large hexagonal bi- and trilayer graphene single crystals with varied interlayer rotations. *Angew. Chem. Int. Ed. Engl.* **53**, 1565-1569 (2014).
4. Carozo, V. *et al.* Raman signature of graphene superlattices. *Nano Lett.* **11**, 4527-4534 (2011).
5. Yeh, C.-H. *et al.* Probing interlayer coupling in twisted single-crystal bilayer graphene by Raman spectroscopy. *J. Raman Spectrosc.* **45**, 912-917 (2014).
6. Lu, C. C. *et al.* Twisting bilayer graphene superlattices. *ACS Nano* **7**, 2587-2594 (2013).
7. Kim, K. *et al.* Raman Spectroscopy Study of Rotated Double-Layer Graphene: Misorientation-Angle Dependence of Electronic Structure. *Phys. Rev. Lett.* **108**, 246103 (2012).
8. Gupta, A. K., Tang, Y., Crespi, V. H. & Eklund, P. C. Nondispersive RamanDband activated by well-ordered interlayer interactions in rotationally stacked bilayer graphene. *Physical Review B* **82** (2010).
9. Chen, Y. *et al.* Raman mapping investigation of chemical vapor deposition-fabricated twisted bilayer graphene with irregular grains. *Phys. Chem. Chem. Phys.* **16**, 21682-21687 (2014).
10. Shu, H., Chen, X. & Ding, F. The edge termination controlled kinetics in graphene chemical vapor deposition

growth. *Chem. Sci.* **5**, 4639-4645 (2014).
11. Artyukhov, V. I., Liu, Y. Y. & Yakobson, B. I. Equilibrium at the edge and atomistic mechanisms of graphene growth. *Proc. Natl. Acad. Sci. U. S. A.* **109**, 15136-15140 (2012).
12. Nguyen, V. L. *et al.* Seamless Stitching of Graphene Domains on Polished Copper (111) Foil. *Adv. Mater.* **27**, 1376-+ (2015).
13. Deng, B. *et al.* Wrinkle-Free Single-Crystal Graphene Wafer Grown on Strain-Engineered Substrates. *ACS Nano* **11**, 12337-12345 (2017).
14. Huang, M. *et al.* Highly Oriented Monolayer Graphene Grown on a Cu/Ni(111) Alloy Foil. *ACS Nano* **12**, 6117-6127 (2018).
15. Brown, L. *et al.* Polycrystalline Graphene with Single Crystalline Electronic Structure. *Nano Lett.* **14**, 5706-5711 (2014).
16. Zhang, J. *et al.* Clean Transfer of Large Graphene Single Crystals for High-Intactness Suspended Membranes and Liquid Cells. *Adv. Mater.* **29**, 1700639 (2017).
17. Meyer, J. C. *et al.* The structure of suspended graphene sheets. *Nature* **446**, 60-63 (2007).
18. Brihuega, I. *et al.* Unraveling the Intrinsic and Robust Nature of van Hove Singularities in Twisted Bilayer Graphene by Scanning Tunneling Microscopy and Theoretical Analysis (vol 109, 196802, 2012). *Phys. Rev. Lett.* **109**, 196802 (2012).
19. Havener, R. W., Liang, Y. F., Brown, L., Yang, L. & Park, J. Van Hove Singularities and Excitonic Effects in the Optical Conductivity of Twisted Bilayer Graphene. *Nano Lett.* **14**, 3353-3357 (2014).
20. Yin, J. B. *et al.* Selectively enhanced photocurrent generation in twisted bilayer graphene with van Hove singularity. *Nat. Commun.* **7**, 10699 (2016).
21. Xu, Z. W., Li, X. X., Yakobson, B. I. & Ding, F. Interaction between graphene layers and the mechanisms of graphites superlubricity and self-retraction. *Nanoscale* **5**, 6736-6741 (2013).
22. Li, Y., Sun, L., Liu, H., Wang, Y. & Liu, Z. Preparation of single-crystal metal substrates for growth of high-quality two-dimensional materials. *Inorg. Chem. Front.*, 10.1039/D1030QI00923G (2020).
23. Zhao, Y. *et al.* Supertwisted spirals of layered materials enabled by growth on non-Euclidean surfaces. *Science* **370**, 442 (2020).
24. Wang, Z. J. *et al.* Direct Observation of Graphene Growth and Associated Copper Substrate Dynamics by in Situ Scanning Electron Microscopy. *ACS Nano* **9**, 1506-1519 (2015).
