# Supplementary material for: Hetero-site nucleation for growing twisted bilayer graphene with a wide range of twist angles
Source: Nat Commun. 2021 Apr 22;12:2391. doi: 10.1038/s41467-021-22533-1 (PMC8062483; doi:10.1038/s41467-021-22533-1)
Supplement: Supplementary file 3 — Source Data [file 41467_2021_22533_MOESM3_ESM.zip › Source Data File/CFD_simulation source files/Description of the CFD_simulation source files.docx]

| File names | Descriptions |
| --- | --- |
| 1_CFD_setting file.cas | Simulation project file, including grid, boundary conditions, calculation model, solver settings, etc. |
| 2_mass_in.c | Custom boundary module for flow and pump speed control. |
| 3_1_CFD_tp-15s.dat | Simulation result file at *t*_p_ – 15 s. |
| 3_2_CFD_tp-1s.dat | Simulation result file at *t*_p_ – 1 s. |
| 3_3_CFD_tp+1s.dat | Simulation result file at *t*_p_ + 1 s. |
| 3_2_CFD_tp+20s.dat | Simulation result file at *t*_p_ +20 s. |
| *t*_p_: the time when the perturbation was introduced | |
